# The organization of a mental health phoneline in Buenos Aires City: its role to minimize the impact of mental health services disruption amidst COVID-19 pandemic

**DOI:** 10.1192/bjo.2021.813

**Published:** 2021-06-18

**Authors:** David Alejandro Gutnisky, Humberto Persano, Victoria Kugler

**Affiliations:** General Direction of Mental Health

## Abstract

**Aims:**

The main concern of this research is to evaluate the performance of a new Mental Health Phoneline Programme, developed to facilitate access to Mental Health Services and to lower the impact of Mental Health Services disruption due to COVID-19 lockdown. Crisis resolution, new referrals, and patients’ reconnection with their former Mental Health Teams were recorded.

**Method:**

The data obtained from 11,406 calls made to the Mental Health Phone Line from April 14th, 2020 to March 1st, 2021 were analysed. Crisis resolutions, new referrals, and patients’ reconnection with their former Mental Health Teams were calculated.

**Result:**

Of the 11,406 calls registered, 72.2% of them were made by women. Mean age was 50.13 years, SD 18.51; median: 50. There was a significant difference between gender regarding age (males: mean 43.91 years, SD 18.88; females: mean 52.48 years, SD: 15.9), being the males who used the phoneline younger (t:23.75; p <0.000). 54.2 % of the users lived with a significant other. Crisis resolution represented 12.6 % of the sample, request for information 34.4%, psychosocial interventions 47.6% and, reconnection with former Mental Health Team 4.3%. New referrals for treatment were 2.9% of the total calls. Two main negative affects the74.2% of the total affect reported. Anxiety-Fear accounts for 49.3% of reported feelings and depression a 24.9 %.

**Conclusion:**

